# Managing spot blotch disease in wheat: Conventional to molecular aspects

**DOI:** 10.3389/fpls.2023.1098648

**Published:** 2023-02-21

**Authors:** Chandan Roy, Xinyao He, Navin C. Gahtyari, Sunita Mahapatra, Pawan K. Singh

**Affiliations:** ^1^ Department of Genetics and Plant Breeding, Agriculture University, Jodhpur, Rajasthan, India; ^2^ Global Wheat Program, International Maize and Wheat Improvement Center (CIMMYT), Mexico DF, Mexico; ^3^ Crop Improvement Division, ICAR–Vivekanand Parvatiya Krishi Anushandhan Sansthan, Almora, Uttarakhand, India; ^4^ Department of Plant Pathology, Bidhan Chandra Krishi Viswavidyalaya, Mohanpur, West Bengal, India

**Keywords:** *Bipolaris sorokiniana*, disease management, resistance breeding, spot blotch, wheat

## Abstract

Spot blotch (SB) caused by *Bipolaris sorokiniana* (teleomorph *Cochliobolus sativus*) is one of the devastating diseases of wheat in the warm and humid growing areas around the world. *B. sorokiniana* can infect leaves, stem, roots, rachis and seeds, and is able to produce toxins like helminthosporol and sorokinianin. No wheat variety is immune to SB; hence, an integrated disease management strategy is indispensable in disease prone areas. A range of fungicides, especially the triazole group, have shown good effects in reducing the disease, and crop-rotation, tillage and early sowing are among the favorable cultural management methods. Resistance is mostly quantitative, being governed by QTLs with minor effects, mapped on all the wheat chromosomes. Only four QTLs with major effects have been designated as *Sb1* through *Sb4*. Despite, marker assisted breeding for SB resistance in wheat is scarce. Better understanding of wheat genome assemblies, functional genomics and cloning of resistance genes will further accelerate breeding for SB resistance in wheat.

## Introduction

**Figure 1:**
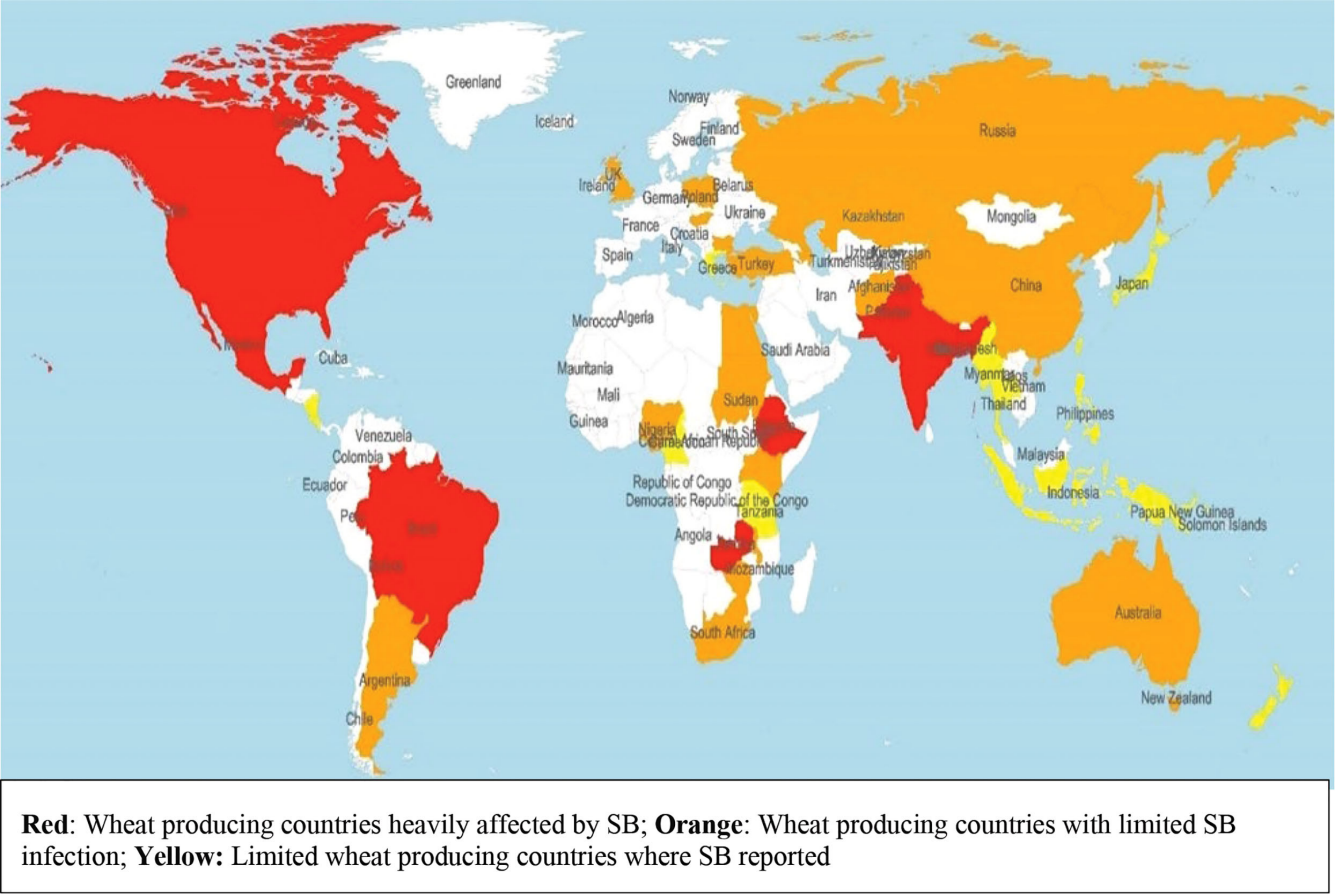
Worldwide distribution of spot blotch of wheat caused by *Bipolaris sorokiniana*.

Spot blotch (SB) caused by the hemibiotrophic fungus *Bipolaris sorokiniana* (teleomorph *Cochliobolus sativus*) syn. *Drechslera sorokiniana*, syn. *Helminthosporium sativum* is the most devastating disease of wheat grown in warm and humid areas. In Eastern Gangetic Plains (EGP) of India, Bangladesh and Nepal, *B. sorokiniana* appears in a complex with *Pyrenophora tritici-repentis* (Died.) Drechs. (anamorph *Drechslera tritici-repentis* (Died.) Shoemaker) responsible for tan spot (TS) and is commonly known as Helminthosporium leaf blight (HLB) (Duveiller et al., 2005). Occurrence of SB is more frequent in the humid and warmer wheat growing areas of South Asia (SA), Latin America and Africa (He et al., 2022) (**Figure 1**). Globally, the disease appears in approximately 25-million-hectare (mha) areas, out of which 10 mha areas are present in EGP. Besides, wheat grown under subtropical lowland of Bolivia, Brazil and Argentina in Latin America, Tanzania, rainfed areas of Zambia and Madagascar in Africa provides congenial environments for SB (van Ginkel and Rajaram, 1998). Under favorable conditions, the disease may cause yield loss of above 50% (Sharma and Duveiller, 2004), with an average yield loss of 15-20% in SA, and yield loss in the farmer’s field reported up to 16% in Nepal and 15% in Bangladesh (Villareal et al., 1995; Saari, 1998; Duveiller, 2002).

**Figure 2:**
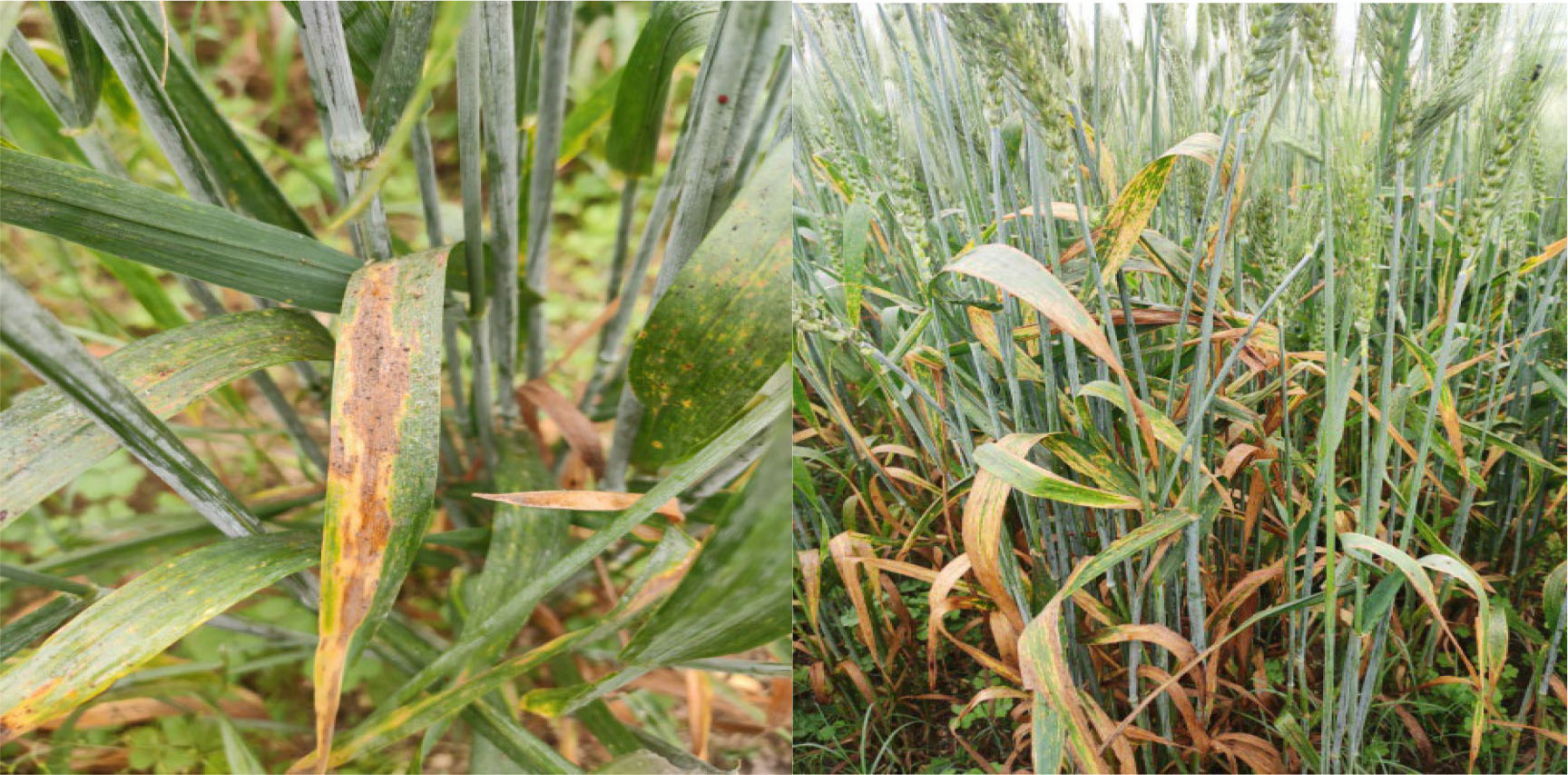
Lesions of spot blotch on infected leaves and plants.

*B. sorokiniana* causes a typical symptom of light brown colored lesions of oval or oblong to elliptical shape on leaves, sheath, nodes or glume of its host plants. Size of the lesion gradually increases, then partial to whole leaf may become chlorotic, turning brown and drying up (**Figure 2**). The pathogen transmits through infected seeds, stubbles and soil, and secondary infection may take place through air. Other than SB, *B. sorokiniana* is also responsible for seedling blight, common root rot, head blight and black point in wheat (Al-Sadi, 2021). No positive association was found among spot blotch, root rot and black point in wheat, indicating that different mechanisms of host resistance exist in different plant parts (Conner, 1990). Cross infection between different species is rarely reported; one such example is isolates collected from wheat root were able to infect barley (Valjavec-Gratian and Steffenson, 1997). A number of articles have been published pertaining to pathogen biology, disease management, breeding and molecular aspects, including genome sequence of *B. sorokiniana*, gene/QTL mapping, marker-assisted selection (MAS) and genomic selection. The present work aims to summarize the most important findings, especially those reported in recent years, in the area of SB management in wheat.

## Pathogen biology

The pathogen produces olive brown mycelia and light grayish colonies on potato dextrose agar (PDA) medium at early stage, which turns into black at later stage. Conidia are brown colored, elliptical, straight or curved multiple celled with 3-9 septa tapering at the ends, measuring 10-28 × 40-120 µm (Acharya et al., 2011). Variability under natural conditions among the *B. sorokiniana* isolates is sufficiently high (Sultana et al., 2018). *Bipolaris sorokiniana* is the asexual stage of the pathogen, which multiplies mostly through conidia. Its sexual stage has not been reported in natural conditions except in Zambia, due to a lack of sexual compatibility between the opposite mating (A, a) types. Under controlled conditions, sexual spores of *B. sorokiniana* were isolated from barley (Zhong and Steffenson, 2001) and recently from wheat in Bangladesh (Sultana et al., 2018).

The fungus can produce toxins like helminthosporol and hydrolytic enzymes that trigger pathogenesis. Prehelminthosporium is the most abundant and active compound produced by *B. sorokiniana*, which damages membrane permeability and affects mitochondrial oxidative phosphorylation and chloroplast photophosphorylation (Kumar et al., 2002). A necrotrophic effector gene *ToxA* interacts with the susceptibility gene *Tsn1* in wheat to initiate disease development (McDonald et al., 2017). *ToxA* was initially identified in *P. tritici-repentis* and then in *Parastagonospora nodorum*, but molecular evidence indicated that the gene in the former was acquired from the latter through horizontal gene transfer (Friesen et al., 2006). *ToxA* was found in *B. sorokiniana* populations from Australia (McDonald et al., 2017), USA (Friesen et al., 2018), India (Navathe et al., 2019b) and Mexico (Wu et al., 2020), with various occurrence frequencies, from 10.2% in Mexico to 86.7% in USA.

Molecular markers may be useful to detect the pathogen on wheat plants before the appearance of visible symptoms, as well as on alternative hosts and volunteer plants. A sequence characterized amplified region (SCAR) marker SCARBS_600_ was developed to diagnose *B. sorokiniana* (Aggarwal et al., 2011). Alternatively, DNA sequence of ribosomal internal transcribe spacer (ITS), β-tubulin gene and translational elongation factor 1-α (EF-1α) can also be used to diagnose this pathogen, as did in a study to detect *B. sorokiniana* in volunteer plants in China (Sun et al., 2015). Using universal rice primers (URP), Aggarwal et al. (2010) successfully grouped 40 *B. sorokiniana* isolates from different geographical origins of India. High level of genetic diversity among the isolates from Brazil and Mexico was reported using UPR markers (Mann et al., 2014).

Draft genome sequences of eight virulent accessions of *B. sorokiniana* from India, Australia, USA and China are currently available (https://www.ncbi.nlm.nih.gov/data-hub/genome/?taxon=45130). A highly virulent isolate BS_112 (GenBank accession number KU201275) from India has a genome size of 35.64 Mb and 10,460 genes were predicted with an average gene length of 435-545 bp and gene density of 250-300 genes/Mb (Aggarwal et al., 2019). A phylogenetic analysis was carried out among 254 isolates of *B. sorokiniana* with global origin using gene sequences of ITS, TEF-1 and GAPDH, and the results indicated the presence of a broad and geographically undifferentiated global population (Sharma et al., 2022).

## Biochemical and molecular events associated with SB infection

*Bipolaris sorokiniana* initially has a biotrophic phase represented by the epidermal invasion and fungal hyphal growth, followed by the necrotrophic phase in the mesophyll cells (Kumar et al., 2002). The germinating conidia penetrates the cuticle and epidermis of wheat plant with the help of an appressorium at its germinating tube, getting entered mostly through the anticlinal cell walls. The levels of sesquiterpene molecule ‘prehelminthosporol’ increases in the extracellular matrix of the cell at the site of apposition, helping pathogen to intrude further into the cell (Jansson and Akesson, 2003).

The first toxin compound known to confer virulence to *B. sorokiniana* was named ‘Victoxinin’ and the second one was ‘sorokinianin’. Another toxin isolated and characterized was ‘bipolaroxin’, which was again structurally a sesquiterpene and had a role in pathogenicity and host selectivity (Jahani, 2005). Helminthosporol was found to enhance the susceptibility of genotypes like Sonalika and CIANO T79. However, it is important to note that helminthosporol and its derivatives are not solely responsible for deciding the susceptibility of a genotype. A multitude of other factors like cell wall apposition, cuticle thickness, leaf anatomy, pathogen specificity, host defense responses etc., are also involved in the pathosystem, making both resistance/susceptibility of the host and virulence/avirulence of the pathogen (Ibeagha et al., 2005; Jahani et al., 2014).

For initial invasion, *B. sorokiniana* produces various cell wall degrading enzymes like glucosidase, cellulases, pectinases, xylanase. Endopolygalacturonase (EPG) loosens the cell wall by cleaving α-(1→4) linkages of the homogalacturonan, an important constituent of the middle lamella of the cell wall (Ridley et al., 2001; Janni et al., 2008). To protect the host cell from EPG, plant cell produces polygalacturonase inhibiting protein (PGIP), which elicits the defense response of a plant by accumulating oligogalacturonides (Ridley et al., 2001). PGIPs have a proven role against the fungal colonization in many dicot species as well as wheat plant against *Bipolaris* (Kemp et al., 2003).

## Epidemiology and host range

Generally, temperature between 16-32°C enables SB development (Acharya et al., 2011), and in Indian subcontinent, the disease predominantly spread when temperature exceeds 26°C as it favors heavy sporulation (Chaurasia et al., 2000). Teleomorph develops in a range of 16-24°C with the optimum temperature of 20°C and can survive up to seven months under natural conditions in Zambia; while the anamorph can survive sufficiently large range of temperature from 4-36°C (Duveiller and Sharma, 2009). High temperature and high relative humidity enhance disease severity, SB outbreak in Brazil occurred when the leaves remain wet for >18 hrs in a day with a mean temperature of >18°C (Reis, 1991). In EGP, leaf wetness period >12hrs due to rainfall or dew coinciding with high temperature and humidity are believed to favor the onset of infection (Duveiller et al., 2005). In addition, delayed sowing of wheat due to the rice-wheat cropping system causes yield loss due to terminal heat stress (Hasan et al., 2021; Narendra et al., 2021); besides, high residual soil moisture and increased duration of leaf wetness due to foggy weather can also increase the disease severity (Duveiller, 2004; Duveiller et al., 2005). The waterlogged condition due to flooding in the Ganges belt sharply declines the conidia viability, and *B. sorokiniana* conidia isolated from soil after August, the monsoon month with high rainfall, becomes non-pathogenic (Pandey et al., 2005). This in turn implies that seeds might be the main source of inoculum in EGP.

SB is a polycyclic disease with the initial sources of inoculum being contaminated seeds, infected soil, straw, volunteer plants and secondary hosts. *B. sorokiniana* has a large host range, and more than 65 graminaceous hosts have been identified in China (Chang and Wu, 1998). Among the cereals, hexaploid wheat and barley are most common hosts, along with durum and emmer wheat, triticale, oats, rice, rye, maize, pearl millet, foxtail millet and several grass species like *Phalaris minor*, *Agropyron pectinatum, A. repens*, *Festuca* spp. (Gupta et al., 2017). A list of plant species that harbors *B. sorokiniana* is given in **Table 1**. The three most common species, *Setaria glauca, Echinochloa colonum* and *Pennisetum typhoids* act as a natural harbor of *B. sorokiniana* in EGP (Pandey et al., 2005). In rice-wheat cropping system, rice plants may serve as a host for the pathogen (Acharya et al., 2011); but in eastern India the source of primary inoculum is still debatable, with infected seeds and weeds being the most probable inoculum sources (Neupane et al., 2010).

**Table 1:** Host species of *B. sorokiniana* (Modified from Manamgoda et al., 2014).

Family	Species group	Species
Poaceae	Cultivated species	*Triticum aestivum, T. durum, Hordeum vulgare, Secale cereale, Tribulus terrestris, Zea mays, Oryza sativa, Eleusine coracana*
	Wild species and grasses	*Aegilops cylindrica, Agropyron buonapartis, A. ciliare, A. cristatum, A. distichum, A. repens, A. trachycaulum var. trachycaulum, A. trachycaulum var. unilaterale, Agrostis capillaries, A. gigantea, A. palustris, Agrostis sp., A. stolonifera var. palustris, Alopecurus pratensis, Aneurolepidium chinense, Arrhenatherum elatius, Avena byzantina, A. sativa, Brachiaria plantaginea, Bromus inermis, B. japonicus, B. marginatus, B. uniloides, B. willdenowii, Buchloe dactyloides, Chloris virgata, Cynodon dactylon, C. transvaalensis, Dactylis glomerata, Dendrobium sp., Digitaria sanguinalis, Echinochloa crus-galli, Ehrharta calycina, E. indica, Elymus breviaristatus, E. canadensis, E. riparius, E. sibiricus, E. trachycaulus, E. virginicus, Elytrigia intermedia, E. repens, Eragrostis cilianensis, Festuca arundinacea, F. ovina, F. pratensis, F. rubra, Holcus lanatus, Hordeum brevisubulatum, H. jubatum, H. leporinum, H. murinum, H. sativum, Hystrix patula, Leymus angustus, L. cinereus, Lolium multiflorum, L. perenne, Microlaena stipoides, Microstegium vimineum, Miscanthus sinensis var. zebrinus, Panicum dichotomiflorum, P. lacromanianum, P. virgatum, Paspalum notatum, Pennisetum clandestinum, Phalaris arundinacea, P. canariensis, Phleum pratense, Phleum* sp., *Poa annua, P. pratensis, P. sylvestris, P. trivialis, Psathyrostachys juncea, Roegneria hirsuta, Saccharum* sp., *Secale montanum, Setaria viridis, Sporobolus vaginiflorus, Stenotaphrum secundatum, Tribulus terrestris, T. secale, Triticum* sp., *T. sphaerococcum, T. vulgare, Zizania aquatica, Z. palustris*
Non-poaceae	*-*	*Allium* sp., *Helianthus annuus, Calluna vulgaris* (Alliaceae), *Taraxacumkok-saghyz* (Compositae), (Ericaceae), *Cicer arietinum, Lablab purpureus, Medicago sativa, Phaseolus vulgaris* (Fabaceae), *Linum usitatissimum* (Linaceae), *Lythrum salicaria* (Lythraceae) *Broussonetia papyrifera* (Moraceae), *Fagopyrum esculentum* (Polygonaceae), *Amaranthus viridis, Glycine max*.

## Disease management strategies

Management of SB through resistant varieties is the most economical and environment-friendly approach, which, however, is compromised by a lack of highly resistant varieties. Under this circumstance, cultivation of resistant varieties may be supplemented with other strategies like adjusting sowing time and fungicides application to reduce the SB severity in disease prone areas. Details of these strategies are described below.

### Chemical control

Seed treatment is always useful to avoid the introduction of additional inoculum. Seed treatment with carboxin or thiram can effectively reduce the load of primary inoculum, especially for seeds with more than 20% infection rate (Mehta et al., 1992). However, seed treatment alone cannot guarantee low spot blotch infection in field (Singh et al., 2014) and foliar fungicidal application is often indispensable. Triazole fungicides like propiconazole, tebuconazole, flutriafol, iprodione, prochloraz, and triadimenol are effective in SB management, e.g., application of Opus (epoxiconazole) significantly reduced the disease severity and maintained it below 10% (Sharma and Duveiller, 2006). In addition, application of Carbendazim and Azoxystrobin has also shown efficacy in controlling the disease (Navathe et al., 2019a). Applying both seed treatment and foliar spray can further reduce the disease, especially when the latter is conducted twice, e.g., upon the appearance of initial infection symptom and 10-20 days later (Singh et al., 2014; Navathe et al., 2019a). Systemic fungicides are more effective than contact fungicides, but the recommended dose should be strictly followed to avoid the emergence of resistant pathotypes against fungicides.

Despite its effectiveness in SB management, fungicidal application increases the cost of cultivation and brings environmental hazardousness. An estimated cost of 153.5 million AUD including application cost is required for fungicides to control wheat diseases in Australia (Murray and Brennan, 2009). Besides, excessive use of systemic fungicides may lead to changes in pathogenic virulence and development of resistance against fungicides. Fungicide resistance has been reported for leaf blight related pathogens *P. tritici-repentis* causing tan spot (Sautua and Carmona, 2021). This is due to the directional selection on pathogen population for resistant pathotypes.

Poor nutrient management is reported to be associated with higher SB infection (Sharma and Duveiller, 2004). Appropriate nitrogen application reduces SB infection, and balanced application of nitrogen along with phosphorus and potassium can further reduce SB severity (Sharma et al., 2006a). Exogenous use of silicon significantly reduces SB severity by increasing the incubation period of the pathogen in wheat (Domiciano et al., 2010). Similarly, application of silver nanoparticles significantly reduced SB infection in wheat, with the induced lignin deposition in vascular bundles (Mishra et al., 2014).

### Cultural practices

Crop rotation is an effective practice for minimizing the primary inoculum load of *B. sorokiniana* in wheat, and the rotation systems of wheat-rice, wheat-oat, wheat-sunflower, and wheat-soybean may be adopted instead of wheat monoculture. Crop rotation provides time to decompose the infected stubble in the field, which helps in improving soil health. Crop residue burning reduces inoculum load up to 90%; but is associated with environmental hazardousness. Alternatively, tillage can be adopted to minimize the load of primary inoculum from the infected stubble. But this may delay the sowing of wheat crop particularly in rice-wheat cropping system, exposing wheat to SB conducive conditions. Zero tillage, minimum tillage or use of happy seeder are alternatives to traditional tillage practices in rice-wheat cropping system (Acharya et al., 2011). Zero tillage facilitates the sowing 10-15 days earlier which helps in escaping the terminal heat stress and results in yield gain by 10-25% in EGP (Joshi et al., 2007c; McDonald et al., 2022). Early sowing is effective in reducing SB, subjected to selection of suitable variety for early sowing as 1) the genotypes must have capacity to tolerate high temperature at early crop growth stage; 2) proper management of foliar blight diseases; as sometimes higher leaf bight incidence was observed upon early sowing due to high residual moisture and humidity (Duveiller, 2004). Therefore, judicial selection of resistant variety is required to minimize the trade off in yield gain by early sowing and higher incidence of leaf blight. In a study, PBW 343, HUW 234 and HUW 468 were found suitable for growing under zero tillage practices (Joshi et al., 2007a).

### Disease resistance

Growing resistant variety is the most effective method of managing crop disease. It is noteworthy that commercial varieties are moderately resistant to susceptible, and such varieties could be heavily infected under SB conducive environment. An early study by Sharma and Dubin (1996) showed increased resistance using multiline mixture resulted in reduction of area under disease progress curve (AUDPC) up to 57% and increased yield up to 8.6% than the component lines. Evaluation of wheat germplasm under different agro-climatic conditions has led to the identification of resistant genotypes. Genotypes SW 89-5193, SW 89-3060 and SW 89-5422 were resistant with 3.9, 2.6 and 3.5% reduction in grain weight, respectively, due to HLB, compared to 33% and 27.6% loss in susceptible cultivars BL 1135 and Sonalika, respectively (Sharma et al., 2004a). Sharma et al. (2004b) reported that SW 89-5422, Yangmai-6, Ning 8201, Chirya 7, Chirya 1 and CIGM90.455 were HLB resistant. These genotypes have been used in developing resistant lines. Furthermore, increasing the level of resistance in new varieties will be effective in managing SB in disease prone areas. Near immune line has been developed from the crosses between resistant genotypes (Kumar et al., 2019); yet further field evaluations are needed to confirm the stability of their resistance as well as their performance in other traits for possible release as varieties.

## Genetics of disease resistance

Quantitative nature of SB resistance is predominantly reported in wheat (Singh et al., 2018; Bainsla et al., 2020; He et al., 2020), but reports are also available for major gene(s) governing SB resistance. Single dominant gene was postulated in the resistant genotypes Chirya 3 and MS#7 when they were crossed with common susceptible parent BL1473 (Neupane et al., 2007). Likewise, single dominant gene was reported in the resistant genotype DT 188, whereas digenic dominant resistance was reported in the lines E5895, HD 1927 and Motia (Adlakha et al., 1984). More genes were estimated in other resistant varieties, e.g., 2-3 genes in Gisuz, Cugap, Chirya 1 and Sabuf (Velazquez-Cruz, 1994), and three genes in Acc.8226, Mon/Ald and Suzhoe#8 (Joshi et al., 2004b). Populations in these two studies have already began to show a pattern similar to polygenic segregation, implying that most resistant sources are governed by multiple genes with minor effects, which increases the chances of deriving transgressive segregants in the progenies (Singh et al., 2018; He et al., 2020). The magnitude of heritability (*h^2^*) for SB resistance varied greatly in different studies, e.g., from 0.21 to 0.64 in Sharma et al. (2006b) and 0.85 to 0.89 in He et al. (2020), whereas most studies exhibited moderate to high heritability, providing a good opportunity to select resistant genotypes in breeding programs.

### Detection of quantitative trait loci (QTL)

Quantitative disease resistance slows down the disease development by increasing the latency period, though, does not always show a clear-cut difference from qualitative resistance conferred by gene-for-gene interaction (Krattinger and Keller, 2016). Most of the QTLs for SB resistance were detected using bi-parental mapping population (**Table 2**), and four major QTLs have been designated as *Sb1* through *Sb4*. *Sb1* was mapped on chromosome arm 7DS, being co-located with *Lr34* (Lillemo et al., 2013) that has been cloned and encodes an ABC (ATP binding cassette) transporter (Krattinger et al., 2009). *Sb2* was detected on chromosome arm 5BL (Kumar et al., 2015), and proposed to be the same locus earlier mapped as *QSb.bhu-5B* in Yangmai 6 (Kumar et al., 2009). Later, *Sb3* was identified in a winter wheat resistant line 621-7-1, being located on chromosome arm 3BS flanked by the markers *Xbarc133* and *Xbarc147* (Lu et al., 2016). Recently, *Sb4* was detected and fine mapped on chromosome arm 4BL flanked by the markers *YK12831* and *YK12928* (Zhang et al., 2020). Besides, a necrosis insensitivity gene *tsn1* was mapped on 5BL associated with *ToxA* insensitivity, and selection for genotypes with *tsn1* may confer resistance against *B. sorokiniana* isolates with *ToxA* (Navathe et al., 2019b).

**Table 2:** QTLs/MTAs (PVE of ≥10%) mapped on different chromosomes for spot blotch resistance using linkage mapping and GWAS.

Chromosome	Flanking markers/MTAs		QTL interval (cM)/Marker position (bp)	PVE (%)	References
Linkage mapping					
4B	985312 - 1241652		39.5 – 41.5	13.7	(Gahtyari et al., 2021)
5D	1058378–1048778		38.5–51.5	15.0	
4D	BS00036421_51-1119387		70.49–90.31	12.2	(Roy et al., 2021a)
5A	1067537-2257572		331.49–332.06	10.3	
5A	2341646-Vrn-A1		44.2-48.6	19.4	(He et al., 2020)
5A	987242-IWA4449		174.6-188.2	25.6	
5B	996745-10592866		73.9-77.7	17.2	
5A	Vrn-A1-3064415		175.9–179.4	12.5	(Singh et al., 2018)
5A	1135154-2260918		147.5–148.4	25.1	
7B	wmc758-wmc335		8.6	11.4	(Singh et al., 2016)
Sb2/QSb.bhu-5B/5BL	Xgwm639-Xgwm1043		0.62	42.4	(Kumar et al., 2015)
QSb.cim-3B	990937|F|0–1123330|F|0		2.7	17.6	(Zhu et al., 2014)
QSb.cim-5A	1086218|F|0–982608|F|0		12.1	12.3	
QSb.bhu-2A/2AS	Xgwm425-Xbarc159		8.5	15.2	(Kumar et al., 2010)
QSb.bhu-2B/2BS	Xgwm148-Xbarc91		21.2	23.7	
QSb.bhu-2D/2DS	Xgwm455-Xgwm815		9.0	10.7	
QSb.bhu-5B/5BL	Xgwm067-Xgwm213		9.0	10.7	
QSb.bhu-7B/7BS	Xgwm263-Xgwm255		5.0	10.2	
QSb.bhu-7D/7DS	Xgwm111-Xgwm1168		3.0	39.2	
QSb.bhu-2A/2AL	Xbarc353-Xgwm445		37.4	14.8	(Kumar et al., 2009)
QSb.bhu-2B/2BS	Xgwm148-Xgwm374		15.0	20.5	
QSb.bhu-5B/5BL	Xgwm067-Xgwm371		13.2	38.6	
QSb.bhu-6D/6DL	Xbarc175-Xgwm732		30.1	22.5	
Genome wide Association studies (GWAS)					
2A	AX-94710084		764783606	31.3	Kumar et al., 2022
2A	AX-94865722		765138703	32.0	
2A	AX-95135556		764819041	31.7	
2B	AX-95217784		800119910	30.1	
2D	AX-94901587		640297481	31.3	
3B	AX-94529408		719773163	31.8	
4D	AX-94560557		442164847	31.4	
Q.Sb.bisa-1A	S1A_497201550 & S1A_497201682		497200000	18.8	Tomar et al. (2021)
Q.Sb.bisa-1B	S1B_636840957		636840000	16.3	
Q.Sb.bisa-1D	S1D_89835681		89840000	24.0	
Q.Sb.bisa-2A	S2A_703111105- S2A_704446408		703110000-704450000	22.8	
Q.Sb.bisa-2B	S2B_419320960-S2B_423836280		419320000-423840000	30.7	
Q.Sb.bisa-4A	S4A_725538462 & S4A_725660945		725540000-725660000	23.0	
Q.Sb.bisa-5B	S5B_682958475 & S5B_683240735		682960000-683240000	31.4	
Q.Sb.bisa-6D	S6D_6395796-S6D_7194112		640000-7190000	20.2	
3A	1085203		595935042	17.7	(Bainsla et al., 2020)
3A	1220348		598916422	13.2	
4A	991620		658343324	12.3	
5A	100177527		3319047	17.6	
5A	5411867		586600348	17.7	
5A	998276		569660176	10.6	
1A	S1A_582293281		582293281	10.0	(Jamil et al., 2018)
1D	S1D_479711997		479711997	11.0	
2A	S2A_16824871		16824871	10.0	
2D	S2D_389463371		389463371	10.0	
3A	S3A_180419285		180419285	13.0	
3A	S3A_741852990		741852990	10.0	
4B	S4B_554842477		554842477	13.0	
5A	S5A_50162259		50162259	11.0	
5B	S5B_501480761		501480761	10.0	
5B	S5B_502451973		502451973	10.0	
5B	S5B_503326206		503326206	10.0	
5B	S5B_504309131		504309131	12.0	
5B	S5B_508031185		508031185	10.0	
5B	S5B_513590441		513590441	11.0	
5B	S5B_528990456		528990456	12.0	
6B	S6B_9296088		9296088	12.0	
7A	S7A_483878120		483878120	10.0	
7B	S7B_749474154		749474154	14.0	

**Figure 3:**
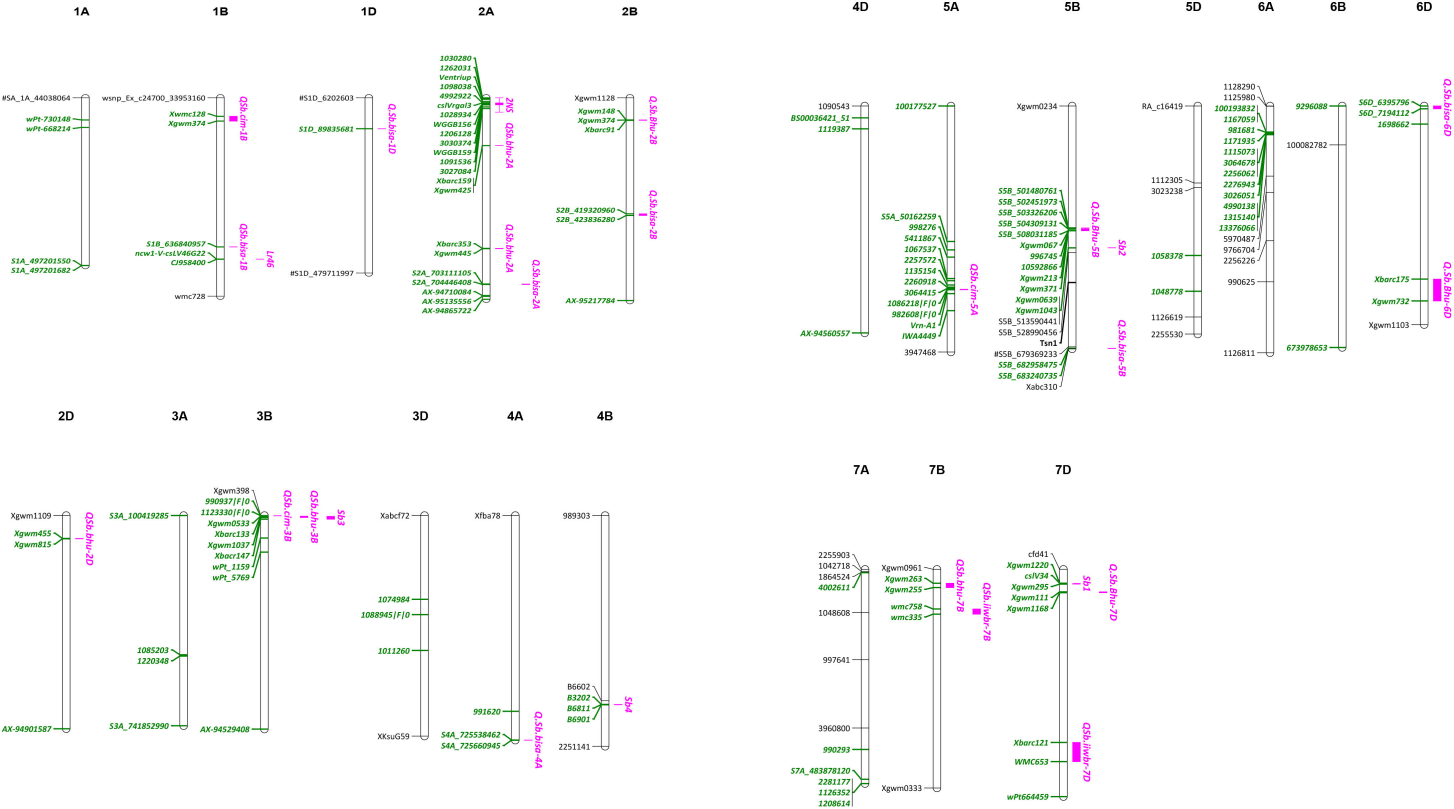
An integrated map showing genes and QTLs for SB resistance and their flanking markers (green color).

Genome wide association studies (GWAS) have been widely performed to detect SB resistance QTLs in wheat. In a study using 566 spring wheat landraces, Adhikari et al. (2012) reported four genomic regions on 1A, 3B, 7B and 7D that were associated with SB resistance. One of the markers, wPt-1159 on 3B, was also associated with resistance to powdery mildew, yellow rust and grain yield (Crossa et al., 2007), and thus could be more useful in breeding. A GWAS on 528 spring wheat landraces with global origin reported 11 significant markers on chromosomes 1B, 5A, 5B, 6B and 7B with phenotypic variation explained (PVE) ranging from 0.14 to 5.80% (Gurung et al., 2014). Recently, 25 significant marker-trait associations (MTA) were identified in a panel of 301 Afghan wheat lines, being located on chromosomes 1A, 1B, 1D, 2B, 2D, 3A, 3B, 4A, 5A, 5B, 6A, 7A, and 7D with PVE ranging from 2.0-17.7% (Bainsla et al., 2020). Major challenges are faced by wheat breeders due to most identified QTLs/MTAs are of minor effects and have no diagnostic markers. Till date, diagnostic markers are available only for *Sb1* (*Lr34*) (Krattinger et al., 2009) and *tsn1* (Faris et al., 2010). A list of QTLs/MTAs for SB resistance is shown in **Table 2**, and an integrated map indicating major genes, QTLs and MTAs on different wheat chromosomes is shown in **Figure 3**.

### Traits associated with SB resistance

Association of SB resistance with plant height and heading date has been well established, with high stature and late maturity often associated with low SB severity (Singh et al., 2015; He et al., 2020). This is mostly due to disease escape mechanisms; though, the possibility of tight linkage of *Rht* and *Vrn* genes with SB resistance genes cannot be ruled out (Zhu et al., 2014; He et al., 2020). Nevertheless, such linkages can be broken, as early and short lines with good SB resistance have been identified in some studies (Joshi et al., 2002; Sharma and Duveiller, 2004; Joshi et al., 2007d). Such early lines are especially important for SA, where terminal heat stress and SB are major yield constraints.

Leaf orientation influences the plant micro-climate, particularly temperature and humidity, through regulating evapotranspiration, and germplasm with erect to semi-erect leaves often showed good SB resistance (Joshi and Chand, 2002). Leaf tip necrosis associated with SB resistance serves as a good morphological marker (Joshi et al., 2004a). Stay-green (SG) genotypes are photosynthetically more active under biotic and abiotic stress conditions, and a positive correlation of SG with SB resistance was reported (Joshi et al., 2007b).

## Breeding for disease resistance

### Sources of SB resistance

Green revolution has resulted in the development of cultivars of semi-dwarf, fertilizer responsive and wider adaptability, enabling the cultivation of wheat in non-traditional areas, including the humid and hot regions with severe SB epidemics. Significant genetic variation for SB resistance was observed among the genotypes evaluated in India, Nepal and Bangladesh (Sharma et al., 2004a; Joshi et al., 2007d). Large-scale SB screening programs were initiated in the late 1980s when the disease became a major threat to wheat production in SA (Duveiller and Sharma, 2012). Initially, resistant sources were identified from Latin American particularly Brazilian germplasm like BH 1146, CNT 1, Ocepar 7, as well as from China like Shanghai 1 to 8, Suzhoe 1 to 10, Wuhan 1 to 3, Ning 8201, Longmai 10 and Yangmai #6 (van Ginkel and Rajaram, 1998). Using such lines as resistant donors, promising genotypes were developed at CIMMYT-Mexico, which exhibited good resistance against SB when tested in Bolivia, Nepal, India and Bangladesh (Sharma et al., 2004b; Sharma and Duveiller, 2007). Recent large-scale germplasm screening activities involve a work on screening 19,460 accessions from Indian national gene bank under field conditions, and 868 accessions were found to be resistant to moderately resistant (Kumar et al., 2016). Further screening of unexplored germplasm from gene bank has identified near immune response in the genotypes EC664204, IC534306 and IC535188 (Kumar et al., 2022).

Wild relatives are a rich source of SB resistance. The 2NS chromosome segment transferred from *Ae. ventricose* has been associated with resistance against wheat blast (Singh et al., 2021; Roy et al., 2021b), rusts (Helguera et al., 2003), cereal cyst nematode (Jahier et al., 2001) and lodging (Singh et al., 2019), and recently it was also associated with SB resistance (Juliana et al., 2022b). *Thinopyrum curvifolium* (Mujeeb-Kazi et al., 1996; van Ginkel and Rajaram, 1998) and synthetic hexaploid wheat derived from crosses between *T. turgidum* and *Aegilops tauschii* (Mujeeb-Kazi et al., 2007) serve as additional resistant sources. Good examples are Chirya genotypes derived from wide hybridization and exhibited good SB resistance, like Chirya 1, Chirya 3 and Chirya 7 (Sharma et al., 2007; Joshi et al., 2007b). High proportion of SB resistance was reported among the synthetic hexaploids evaluated under controlled conditions (Lozano-Ramirez et al., 2022). However, genotypes with high level of field SB resistance are scarce, a major limitation in the progress of breeding program.

### Development of resistant genotypes

Breeding for resistant genotypes through crossing programs were started in 1980s in CIMMYT, Mexico and still this center is playing an important role in global SB resistance breeding. Ever since 2009, a special nursery was formed as CSISA-SB (presently known as Helminthosporium Leaf Blight Screening Nursery, HLBSN), comprising high yielding SB resistant genotypes for testing over the different countries in SA, Africa and Latin America (Singh et al., 2015). Testing of the 4^th^ CSISA-SB nursery at seven locations in Mexico, India and Bangladesh identified two stably resistant lines (CHUKUI#1 and VAYI#1) consistent over the locations (Singh et al., 2015).

The progress in achieving genetic gain for SB resistance is slow, due to reasons like quantitative inheritance, moderate heritability, strong genotype × environmental interaction, and high variability of the pathogen over time. Phenotypic selection is often associated with confounding traits, and molecular markers can be used to assist the pyramiding of resistance QTLs and selection for SB resistant genotypes (He et al., 2020). Superior genotypes have been developed from a marker assisted backcross program *via* transferring *Qsb.bhu-2A* and *Qsb.bhu-5B* from Chirya 3 and *Qsb.bhu-2A* from Ning 8201 into the genetic background of HUW 234 (Vasistha et al., 2015). Increased resistance up to near immunity could be obtained by stacking effective QTLs from multiple donors (Kumar et al., 2019). However, such QTL stacking could be compromised by QTL × QTL interaction, as demonstrated by Kumar et al. (2019) in a cross between “Yangmai#6” and “Chirya#3”, where QTLs on 6D and 7D have a masking effect on each other. This highlights the importance of understanding the mode of action (additive, dominant, or epistatic) of the QTLs to be utilized in breeding.

Genomic selection can improve the efficiency of breeding program by reducing phenotyping cost, time and increasing selection intensity and genetic gain. Studies on genomic selection for SB in wheat are limited, and a successful example was reported by Juliana et al. (2022a), where genomic selection showed significantly higher accuracy than the fixed effect model using few selected markers. However, there is a long way to go for genomic selection to completely replace phenotypic selection in wheat breeding.

## Biotechnological approaches

Gene silencing through RNAi is a powerful tool for controlling insects, nematodes, viruses, fungal diseases like powdery mildew, and rusts (Qi et al., 2019). Utilization of RNAi in functional genomic analysis in *B. sorokiniana* was reported by Leng et al. (2011), and it can be further explored in SB pathogenesis and resistance breeding in wheat.

Liu et al. (2012) developed a new mapping strategy combining bulk segregant analysis and RNA-Seq called ‘BSR-Seq’, where transcripts are sequenced from extreme bulks, being a potential technique for marker discovery in large polyploid genome like wheat. Using BSR-Seq, five SB-resistance associated transcripts were identified on 5B and 3B chromosomes and their potential role in SB resistance were inferred (Saxesena et al., 2022).

Transgenic lines expressing foreign genes proved to be a potential approach to control insect and diseases in several crop plants. Examples include the heteroexpression of *PvPGIP2* (Janni et al., 2008) and overexpression of *TaPIMP1* and *TaPIMP2* (Zhang et al., 2012; Wei et al., 2017) in transgenic wheat lines that enhanced the resistance against *B. sorokiniana*. However, government regulations on transgenic development are major concerns for researchers.

Additional techniques like genome editing (Zhang et al., 2017) and Eco-Tilling (Ajaz et al., 2021) have been increasingly utilized in wheat, having great potential to contribute to SB resistance breeding in near future.

## Conclusion

Spot blotch is a disease of concern in warmer wheat growing areas of South Asia, Latin America and Africa. Most of the commercially grown cultivars are moderately resistant to susceptible and are subjected to significant yield losses under conducive climatic conditions. An integrated disease management strategy involving cultural practices, chemical control, resistant cultivars, etc., is needed to combat the disease. In addition, modern biotechnology brings new tools for the rapid and efficient development of resistant cultivars in wheat.

## Author contributions

CR, XH, and PS conceptualized the manuscript. CR drafted the first version. NG and SM collected information and improved the first draft. XH and PS edited and approved the submitted version. All authors contributed to the article and approved the submitted version.
